# Salivary immunity and lower respiratory tract infections in non-elite marathon runners

**DOI:** 10.1371/journal.pone.0206059

**Published:** 2018-11-21

**Authors:** Elisabet Cantó, Emma Roca, Lidia Perea, Ana Rodrigo-Troyano, Guillermo Suarez-Cuartin, Jordi Giner, Anna Feliu, Jose Manuel Soria, Lexa Nescolarde, Silvia Vidal, Oriol Sibila

**Affiliations:** 1 Experimental Immunology, Institut de Recerca, Hospital Sant Pau, Barcelona, Spain; 2 Biomedical Research Insitute Sant Pau (IIB-Sant Pau), Barcelona, Spain; 3 Universitat Politècnica de Catalunya, Barcelona, Spain; 4 Respiratory Department, Hospital de la Santa Creu i Sant Pau, Autonomous University of Barcelona, Barcelona, Spain; 5 Unit of Genomic of Complex Diseases, Barcelona, Spain; University of the Pacific, UNITED STATES

## Abstract

**Rationale:**

Respiratory infections are common after strenuous exercise, when salivary immunity may be altered. We aim to investigate changes in salivary immunity after a marathon and its relationship with lower respiratory tract infections (LRTI) in healthy non-elite marathon runners.

**Methods:**

Forty seven healthy marathon runners (28 males and 19 females) who completed the 42.195 km of the 2016 Barcelona marathon were studied. Saliva and blood samples were collected the day before the marathon and two days after the end of the race. Salivary IgA, antimicrobial proteins (lactoferrin, lysozyme) and chemokines (Groα, Groβ, MCP-1) were determined using ELISA kits in saliva supernatant. Blood biochemistry and haemogram were analyzed in all participants. The presence of LRTI was considered in those runners who reported infectious lower respiratory tract symptoms during a minimum of 3 consecutive days in the 2 weeks after the race.

**Results:**

Eight participants (17%) presented a LRTI during the 2 weeks of follow-up. Higher lysozyme levels were detected after the race in runners with LRTI when compared with those without infection. A decrease in salivary lysozyme, Groα and Groβ levels after the race were observed in those runners who did not develop a LRTI when compared to basal levels. Salivary Groα levels correlated with basophil blood counts, and salivary lysozyme levels correlated with leukocyte blood counts.

**Conclusions:**

LRTI are common after a marathon race in non-elite healthy runners. Changes in salivary antimicrobial proteins and chemokines are related to the presence of LRTI and correlate with systemic defense cells, which suggest an important role of salivary immunity in the development of LRTI in non-elite marathon runners.

## Introduction

The number of non-elite runners participating in marathons had increased dramatically in the last ten years [1]. Several studies have demonstrated that marathons and ultramarathons may alter immune function and increase the risk of respiratory tract infections [2–4]. Within two weeks of completing such strenuous exercise, the risk of infection increased 100% to 500% [5] and around 25% of finishers reported respiratory symptoms [3].

Salivary Immunoglobulin A (sIgA) is the most common antibody on the mucosal surface. Different studies have analyzed changes in sIgA following strenuous exercises and their potential relationship with respiratory symptoms, with conflicting results [6–8]. Other studies have suggested that immune factors present in mucosal secretion, including antimicrobial peptides (AMPs) and immune mediators such as chemokines, changed after prolonged running. An increase in salivary lactoferrin, one of the most abundant AMPs, has been described in participants in a 50 km ultramarathon [9]. Furthermore, chemokines such as Growth-Regulated Oncogene-alpha (Groα or CXCL1), growth-Regulated Oncogene-beta (Groβ) and monocyte chemoattractant protein 1 (MCP-1) have demonstrated an important role in the regulation of local immunity against infections by contributing to the tissue infiltration of leukocytes [10]. Experimental studies showed that serum levels of these inflammatory mediators increased markedly in response to exercise in mice [11]. However, limited data regarding salivary IgA, AMPs, chemokines and the presence of lower respiratory tract infections (LRTI) in marathon runners are available.

We hypothesized that non-elite marathon runner who experienced changes in their salivary immunity would be more prone to developing a respiratory infection. Therefore, our aim was to determine salivary levels of IgA, AMP and inflammatory markers before and after a marathon and their relationship with the development of LRTI.

## Materials and methods

### Subjects

Forty seven healthy non-elite marathon runners (28 males and 19 females) completed the 42.195 km of the 2016 Barcelona Marathon. Mean finishing time was 3 hours and 38 min (±41 min). Study participants were recruited using an announcement in the marathon newsletter that all marathon runners received two weeks prior to the race. Participation was voluntary in all cases. The study was approved by the local ethics committee “Comité Étic de Investigació Clínica de la Fundació de Gestió Sanitaria del Hospital de la Santa Creu i Sant Pau de Barcelona”, project number IIBSP-SUMMIT-2016-02. All participants gave signed informed consent.

All runners were asked to report any incidence of respiratory symptoms 2 weeks before and after the race. Runners who reported respiratory symptoms 2 weeks before marathon were excluded from the study. LRTI was defined according the clinical definition proposed by the European Respiratory Society and European Society for Clinical Microbiology and Infections disease [12]: acute illness (present for 21 days or less) characterized by cough as the main symptom, with at least one other respiratory tract symptom (sputum production, dyspnea, wheeze or chest discomfort/pain) and no alternative explanation. In addition, all runners had to report at least one determination of fever (≥38°C) and duration of symptoms must be higher than 3 days in order to exclude those symptoms induced only by strenuous exercise. All runners with suspected LRTI were follow-up until a breakdown of symptoms was reported.

### Saliva and blood collection

Saliva and blood samples were collected one day before the marathon and two days after the end of the race. Saliva was collected into clean, sterile tub, maintained at 4°C before centrifugation at 9500xg for 10 minutes at 4°C. Supernatant was aspirated and 3 different aliquots were frozen at -20°C until use. Three 10 mL blood samples were obtained from the antecubital vein and were transferred immediately (in less than 2 hours) to the laboratory for analysis.

### Saliva analysis

The saliva samples were then thawed and total protein saliva was quantified using the Qubit protein Assay Kit (Thermofisher Scientific). Salivary IgA (Human IgA Platinum ELISA, ebioscience, Affymetrix, Santa Clare, CA), lactoferrin, lysozyme (AssayPro, St. Charles, MO, USA), Groα, Groβ and MCP-1 (Elabscience, Houston, Texas) were measured by ELISA according to the manufacturer’s instructions. The limit of detection for IgA was: 1.6 ng/ml; lactoferrin: 0.625 ng/ml; lysozyme: 0.0781 ng/ml; Groα: 15.63 pg/ml, Groβ: 15.63 pg/ml and MCP-1: 15.63 pg/ml. Data were expressed as the concentration of each molecule relative to total saliva protein concentration [13]. Those determinations of relative salivary IgA concentration with a ratio higher than 1 were excluded of the study due to possible contaminations [14]. All determinations were performed in triplicate.

### Blood analysis

Blood samples were centrifuged at 800xg at 4°C for 10 min in a bench centrifuge. Supernatant (plasma) was aliquoted and stored on dry ice until all samples were frozen at -80°C. Sodium and potassium plasma concentrations were determined using ion selective electrodes with an ionized sodium/potassium analyzer KNA1 (Radiometer, Copenhagen, Denmark). Creatinine and urea were measured using an AU5800 analyzer (Beckman Coulter, Hospitalet de Llobregat, Spain). C-reactive protein was determined using the AU-5800 Chemistry Analyzer (Bekman Coulter, Miami, FL, USA). Complete blood counts were performed with the Unicel DxH800 automated hematology analyzer (Beckman Coulter, Miami, FL, USA).

### Statistical analysis

Statistical analyses were performed using Graph Pad Prism 5 software. The Kolmogorov-Smirnov test was applied to test the normal distribution of the data. All variables with a normal distribution were reported as mean ± standard deviation (sd). T-test and paired t-test respectively were used for the comparison of independent and related variables with Gaussian distribution. The Wilcoxon test and Mann Whitney test were used for the comparison of related variables and for the comparison of independent variables respectively with non-normally distributed data. Pearson’s and Spearman’s coefficients respectively were used to correlate changes between normal and non-normal distributed variables. Chi-square tests were used for the comparison of frequencies. P values less than 0.05 were considered significant.

## Results

### Study participants

Table 1 shows demographic and laboratory test results prior to the marathon for all participants. Median age was 30 years old, median years of training were around 10 and training hours per week were around 6. All laboratory tests performed were in the normal range.

**Table 1:** Demographic and biochemical charcateristics from marathon runners.

	All runners	Non LRTI	LRTI	p value
**Sex (M/F)**	28/19	21/18	7/1	NS
**Age**	39.0±7.1	39.1±7.4	38.6±5.6	NS
**BMI**	22.6±2.4	22.3±2.3	23.8±2.8	NS
**Years training**	9.7±9.3	10.2±10.0	7.4±4.0	NS
**Hours training/week**	6.5±3.7	6.5±3.9	6.6±2.6	NS
**Glucose (mg/dl)**	84.7±14.4	85.5±15.4	81.0±7.9	NS
**Na (mEq/l)**	139.3±1.6	139.2±1.4	139.8±2.5	NS
**K (mEq/l)**	4.0±1.6	4.0±0.2	4.0±0.3	NS
**Leukocytes %**	6.9±1.6	6.8±1.4	7.6±2.2	NS
% Lymphocytes	34.7±7.5	34.8±6.5	34.1±11.9	NS
% Monocytes	8.3±2.7	8.5±2.9	7.7±1.3	NS
% Neutrophils	53.9±8.5	53.5±7.5	55.8±12.6	NS
% Eosinophils	2.3±1.7	2.4±1.7	1.7±1.5	NS
% Basophils	0.6±0.4	0.7±0.4	0.4±0.1	NS
**Hb (g/dl)**	14.1±1.1	14.5±0.9	14.0±1.2	NS
**Hematocrit (%)**	41.8±3.3	41.6±3.4	42.8±2.9	NS
**Platelets (x10**^**9**^**/l)**	215.8±50.3	214±48	220±62	NS
**CRP (mg/dl)**	1.3±2.2	1.4±2.4	1.0±0.5	NS

BMI, Body Mass Index; Hb, hemoglobin; CRP, C-reactive protein.

LRTI: Lower Respiratory Tract Infection

NS, non significant

No differences between males and females were found in baseline characteristics (data not shown), except for Body Mass Index (BMI), which was higher in males than in females (23.6 ±2.1 vs 20.7 ± 1.4, p = 0.01).

### Lower respiratory tract infections

Eight participants (17%) reported a LRTI during follow-up. No differences in demographics and laboratory findings between infected and non-infected participants were found prior to the marathon (Table 1). All runners with LRTI experienced a breakdown of the symptoms before 14 days of their onset and were fully recovered within one month after the race.

### Salivary IgA

**Fig 1:**
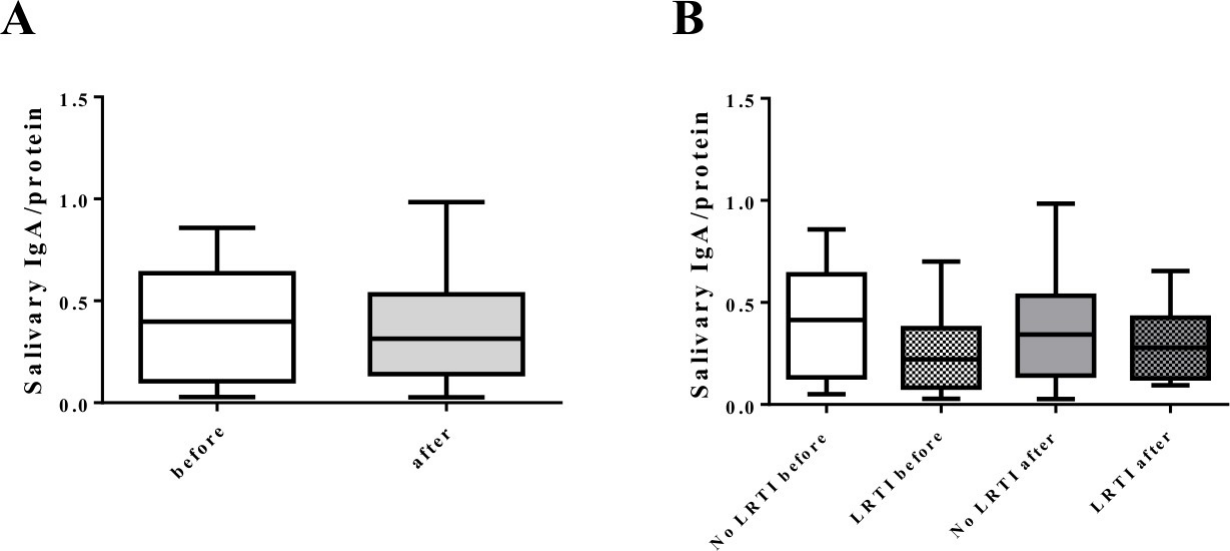
Salivary sIgA from marathon non-elite runners. Salivary sIgA was determined by ELISA as described in Materials and Methods. (A) sIgA content before and after marathon. (B) sIgA content before and after marathon based on the development of LRTI after marathon. Results were showed as the concentration of sIgA relative to total protein content. P values were determined by paired non parametric Wilcoxon t-test.

No differences before and after the marathon were found in total salivary protein concentration (2.34±1.16 mg/ml vs 2.37±0.97 mg/ml, respectively) nor in salivary IgA levels normalized to total salivary protein (0.39±0.25 vs 0.35±0.24) (Fig 1A). In addition, no differences in IgA levels before and after the race were found between infected and non-infected runners (Fig 1B), and no differences were observed between male and females before marathon in salivary IgA/protein.

### Salivary antimicrobial proteins

No differences before and after the race were observed in salivary lactoferrin (0.013±0.01 vs 0.010±0.007) and salivary lysozyme levels (0.0094±0.007 vs 0.0078±0.007). Furthermore, no differences between the sexes were observed before the marathon.

**Fig 2:**
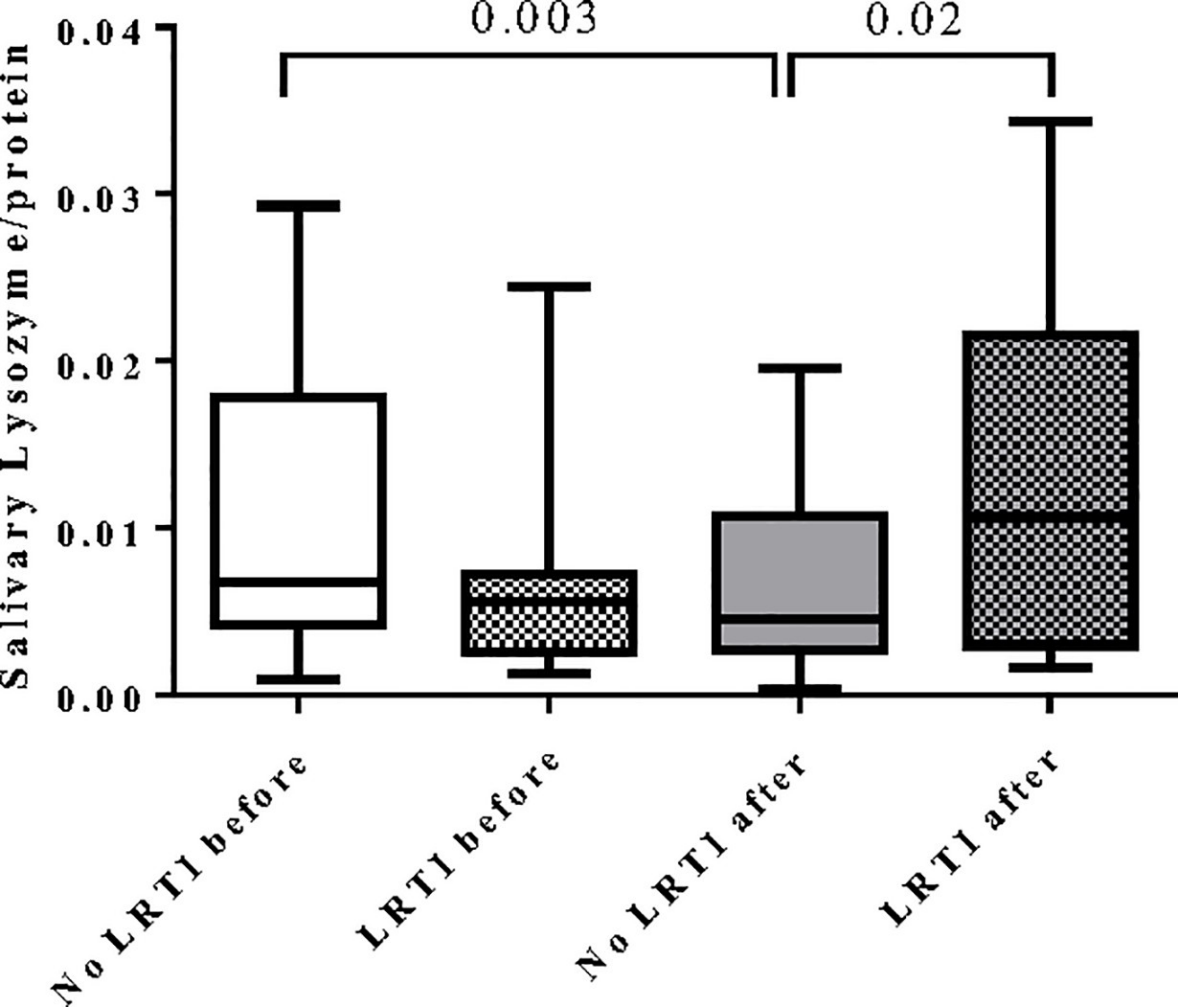
Salivary lysozyme from marathon non-elite runners before and after the marathon. Results were showed as the concentration of lysozyme relative to total protein content. P values were determined by unpaired t-test and paired Wilcoxon test.

When runners with and without LRTI were compared, we observed no differences in salivary lactoferrin levels before and after the race. However, those runners who developed a LRTI showed higher levels of salivary lysozyme after the race compared to those who did not develop it (0.012±0.01 vs 0.006±0.004, p = 0.02). In addition, a decrease in lysozyme levels was observed in runners who did not develop LRTI when levels before and after the race were compared (0.010±0.007 vs 0.006±0.004, p = 0.003) (Fig 2).

### Salivary chemokines

**Fig 3:**
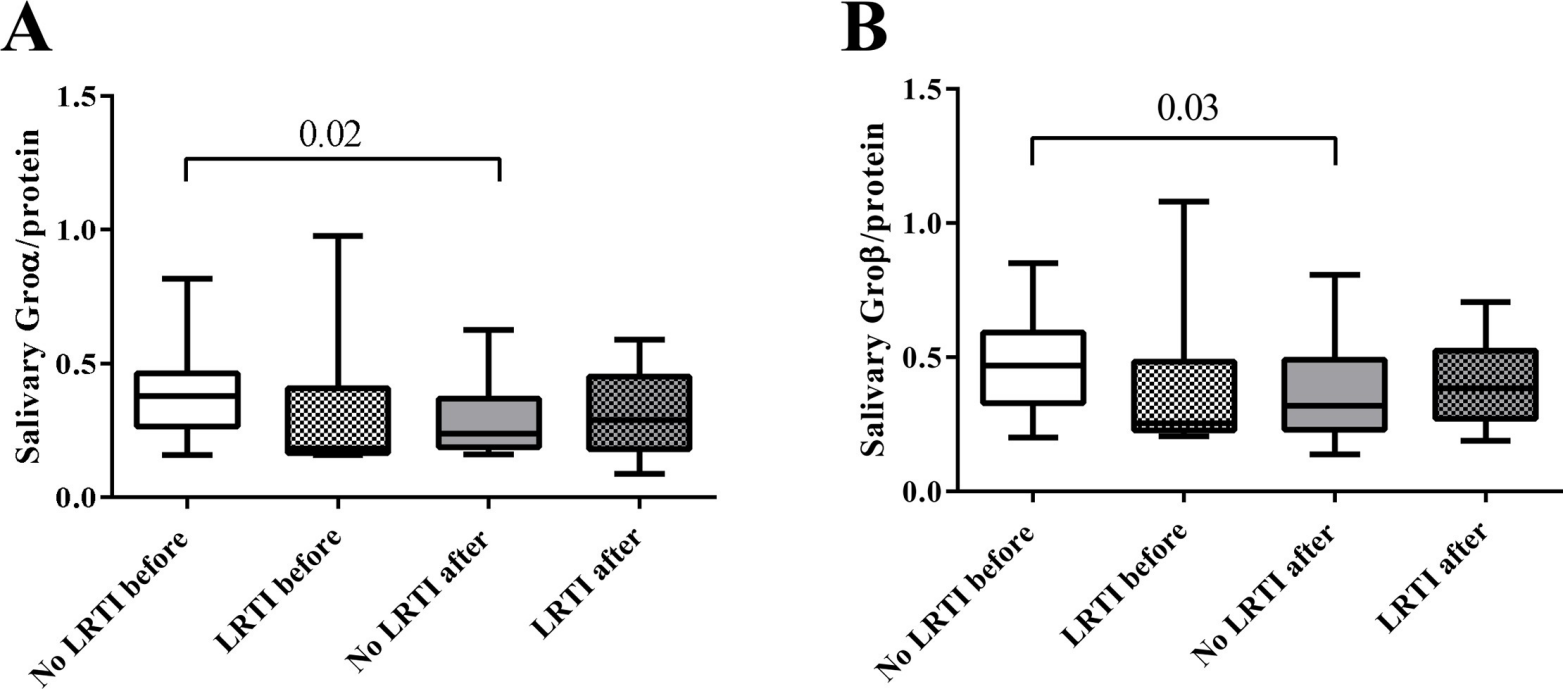
Salivary Groα and Groβ. Salivary (A) Groα and (B) Groβ were determined by ELISA as described in Materials and Methods before and after marathon. P values were determined by unpaired Mann Whitney t-test and paired Wilcoxon test.

No differences before and after the race were observed regarding salivary Groα Groβ and MCP-1. However, runners who did not develop LRTI showed a significant decrease in salivary Groα and Groβ levels after the race (Groα, 0.37±0.15 vs 0.30±0.14, p = 0.02; Groβ 0.47±0.17 vs 0.37±0.18, p = 0.03) (Fig 3A and 3B). No differences in MCP-1 levels were found.

### Systemic correlations

**Fig 4:**
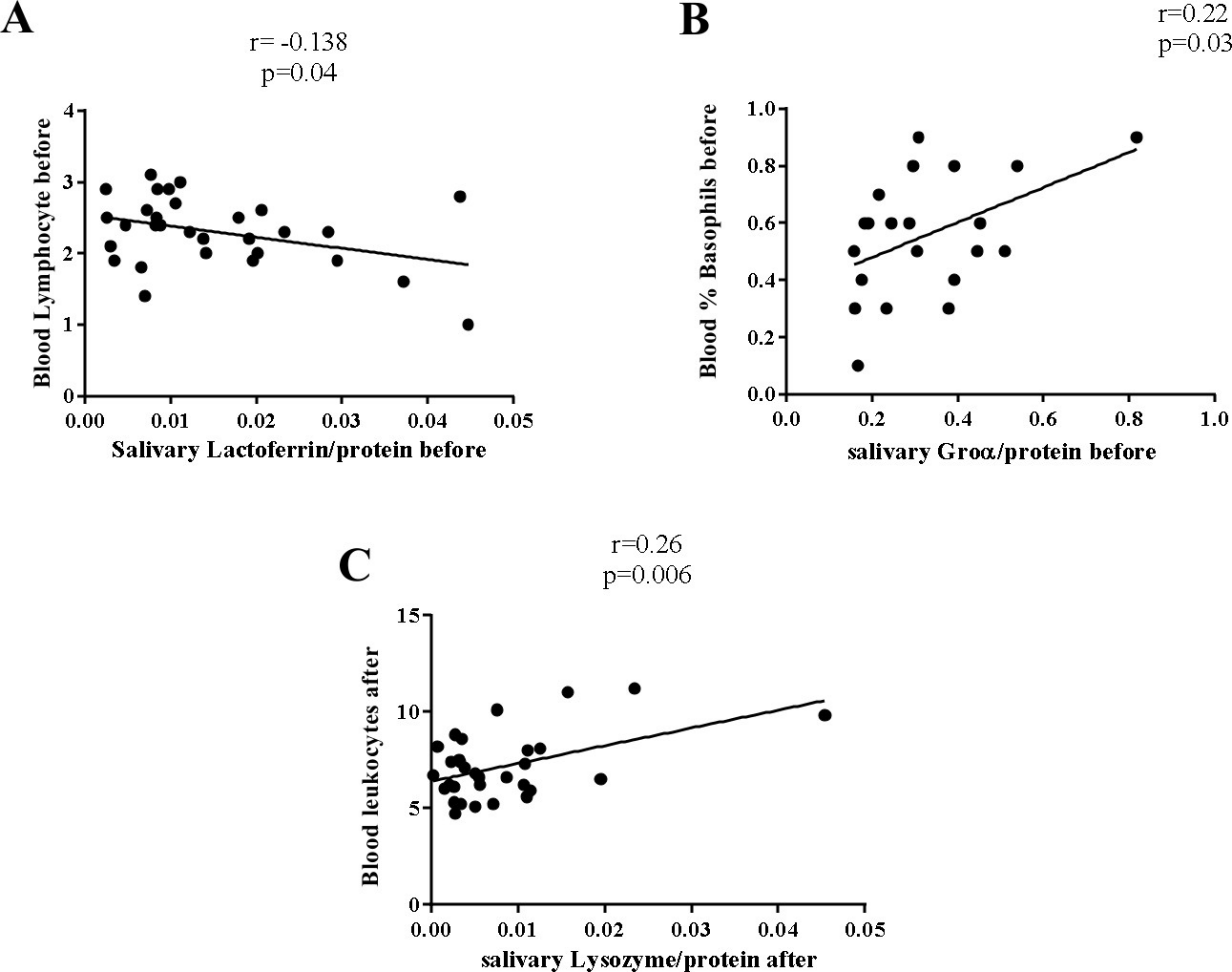
Correlation of salivary lactoferrin, Groα and lysozyme with systemic haematological parameters. **(**A) Negative correlation between salivary lactoferrin with blood lymphocytes, (B) positive correlation between salivary Groα and blood basophils and (C) positive correlation between salivary lysozyme and blood leukocytes, were found. Pearson’s and Spearman’s coefficients were respectively used to correlate changes between normal and between non-normal distributed variables.

Before the race, salivary lactoferrin levels had a weak but statistical significant negative relationship with blood lymphocyte counts (r = -0.13, p = 0.04) and salivary Groα had a weak positive correlation with the percentage of blood basophils (r = 0.22, p = 0.03) (Fig 4A and 4B).

After the race, salivary lysozyme correlated positively with the absolute number of blood leukocytes (r = 0.26, p = 0.006) (Fig 4C).

## Discussion

In our study we observed that LRTI is common in non-elite runners following a marathon and we demonstrated that runners who developed a LRTI had different immunological profile in their saliva. Specifically, a decrease in salivary lysozyme, Groα and Groβ levels was observed in those runners who did not develop a LRTI when levels before and after the race were compared. In addition, higher levels of lysozyme were detected after the race in runners with LRTI compared with those without infection. These findings suggest that immunological status may be important for the development of a LRTI after a marathon.

Several data have been reported reflecting the importance of the immune system in the development of LRTI after strenuous exercise. We observed that 17% of marathon runners develop LRTI symptoms within 2 weeks after marathon, which is concordant to other studies [7,15]. Although the effect of close proximity between runners during the race cannot be ruled out, extreme effort has been shown to alter immune defenses by increasing inflammation in the respiratory tract [16]. Different studies have postulated that a combination of several immune, biochemical and hematological parameters participate in the severe stress on the body resulting from such exercise and that they may be associated with an increased susceptibility to developing infections [17]. Salivary IgA (sIgA) is one of the most widely-studied parameters [7]. Some studies have pointed to the usefulness of sIgA as a noninvasive biomarker of mucosal immunity and LRTI risk [18,19]. However, other studies have suggested that reductions in sIgA cannot be solely responsible for the decline in immune function that may lead to LRTI [20]. In our cohort of runners, we did not observe differences in sIgA as other authors previously showed [21]. We observed that sIgA represented about 40–60% of total salivary protein, which is concordant with previous studies [22,23] but higher than other ones [24]. Most of the studies were carried out few hours after exercise [25,26] and we wanted to evaluate two days after finishing the marathon, since few data regarding the recovery period and its influence on the development of LRTI are available [7].

Salivary antimicrobial proteins (sAMPs) such as lysozyme and lactoferrin protect the respiratory tract from invading microorganisms and have been linked with an increased infection risk in athletes [27]. In our cohort of runners, we observed no differences in the levels of saliva lysozyme before the race. However, runners who did not develop LRTI showed a decrease in lysozyme after the marathon. In contrast, those runners who developed a LRTI showed higher levels of salivary lysozyme after the race compared to those runners who did not develop LRTI. Our findings are in line with other studies showing a decrease in salivary lysozyme immediately after an ultra-marathon, though this decrease was not related to the presence of LRTI [5]. However, other studies did not observe differences in salivary lysozyme levels after a 50 km race [25]. These discrepancies could be the consequence of different levels of effort during exercise. Interestingly we observed an inverse relation between salivary lactoferrin and blood lymphocyte counts before the race, which may be explained by the mobilization of neutrophils to maintain blood homeostasis. Previously, Inoue H et al. [28] described an immediate increase in serum lactoferrin concentrations immediately after running exercise and serum lactoferrin may play an antibacterial role in host defenses before the mobilization of neutrophils into the circulating pool. We also observed a positive correlation between blood leukocyte counts and the content of saliva lysozyme after the marathon. It has been observed that exercise sessions in both humans and animal models resulted in the stimulation of neutrophil degranulation releasing lysozyme from blood neutrophils [29]. All of these findings suggested that systemic alterations may affect the content of saliva lactoferrin and lysozyme in runners and may contribute to the development of LRTI after races. Nevertheless, we cannot exclude a process of dehydration during the marathon, since it has been demonstrated that dehydration decreases saliva antimicrobial proteins [30,31]. Dehydration can therefore affect the content of saliva AMPs, and further studies are needed to better elucidate its importance, though we did not observe differences in protein content before and after the race.

Different chemokines have demonstrated a potential role in the regulation of immune response during exercise [32]. We also found that Groα and Groβ, which are key components in the attraction of neutrophils to the site of inflammation, decreased after the race in runners who did not develop a LRTI. It has been described that daily moderate exercise suppressed Groα [33] and, in experimental models, exercise down regulated multiple inflammatory cytokines and chemokines including Groα and MCP-1 [34,35] and had a systemic anti-inflammatory effect, reducing Groα, Groβ and MCP-1 [36,37]. These findings were observed in runners who did not develop LRTI, suggesting that they had a better inflammatory regulation response that may protect against infection. Therefore, athletic performance is both a stress factor and an adaptive response to exercise that can be modulated by training, reduce inflammation and help to prevent disease. Further studies are needed to better understand the relationship between salivary immunity and systemic inflammation as a key factor in the development of LRTI after exercise.

Interestingly, we observed a positive correlation between blood basophils and salivary Groα level before the race. Sastre B et al, identified basophils as a new player in the status of bronchial inflammation in athletes [38]. We observed that non LRTI runners had a higher percentage of systemic basophils, although differences were not statistically significant.

Our study has several limitations. First, due to the small number of runners included, the results should be validated in other studies before generalizing them. Second, since some data reported the effect of feeding in post exercise saliva AMPs proteins [39] we cannot exclude feeding related affectations in the determinations of AMPs in our cohort of runners. Third, levels of leukocyte subpopulations after the race were measured 48 hours after the marathon and other determinations at 7 or 14 days would be helpful to better characterize systemic response in marathon runners, and it should be an important point to be taken into account in further studies. And fourth, infectious agents that caused LRTI are not described due to the absence of microbiological studies. Further studies including bacterial and viral determinations would be very helpful to clarify this important issue.

In conclusion, non-elite runners who developed a LRTI after a marathon showed a differential profile of saliva IgA, AMPs and chemokines compared to those runners who did not develop infection. Therefore, exercise training and post-marathon recovery would be important in the immunological profile and the risk of developing a LRTI. Further studies are needed to better understand the underlying mechanisms and the impact of salivary immunity and its regulation in the prevention and development of LRTI in non-elite marathon runners.

## Supporting information

S1 File PONE-D-18-11926R3_Data.xlsx. (XLSX)
